# Fatal Hæmorrhage from the Extraction of a Tooth

**Published:** 1842-05

**Authors:** W. A. Roberts

**Affiliations:** Edinburgh


*Fatal Hæmorrhage from the Extraction of a Tooth.* By W. A. Roberts, Esq., Edinburgh.—Mr. C. P., of middle age, rather full-sized body, called upon me on Sunday, the 19th of December, 1841, requesting to have a tooth removed, that had given some uneasiness for a length of time; upon examination, I found the dens sapientiæ of the right side of the lower jaw loose, the crown gone, and removed it without difficulty with a pair of forceps generally used for extracting the temporary teeth of children. It had three small fangs, the anterior one being the longest; the hæmorrhage, nothing more than usual, had ceased ere he left, the alveolus being plugged with lint, wetted with the camphorated spirit of wine. At half past four of the same day, Mr. P. called again, the blood running in a continuous stream, evidently from the anterior alveolus—cleaned it out from the bottom, and filled it up firmly with a strip of lint, pressed down with a curved instrument; when full, applied a compress of cork, fitted to the part and pressed upon firmly by the dens sapientiæ of the upper jaw; likewise securely bandaged the jaw. Ordered astringent lotions, for the hæmorrhage was again checked, the saliva coming away unstained.

At this visit the patient informed me that he had a tooth taken out a few years ago, which was followed by copious hæmorrhage for nearly three days, but was checked by the application of caustic; also that lately his gums had bled to a considerable extent, and for a fortnight at a time. Of all this I was unfortunately ignorant until after three hours had elapsed from the removal of the root. There was nothing indicating any hæmorrhagic tendency at the time I saw him first, and, being a stranger to me, I was consequently not acquainted with the history of his habit of body.

I was sent for early on Monday morning, and found the hæmorrhage had continued without intermission during the night. He had deferred sending for me, unfortunately, as I had requested, supposing the bleeding would stop of itself. I found no coagulum about the mouth, or in what he had spat out, as in ordinary hæmorrhage, the alveolus being as clear as when the stump was first taken away. I put a piece of lunar caustic, the size of a pin's head, into the bleeding alveolus, pressed it down, and plugged with sponge tent and bandaged as formerly. The bleeding was again stopped. Styptics, lotions of kino, and alum, were used with benefit.

For more than an hour after this all appeared safe. In the course of the day, Dr. Hay, of Queen street, the family medical attendant, saw him, and found the hæmorrhage as bad as ever. Applied various styptics without doing much good. On the 21st, Dr. Hay applied the actual cautery without benefit, attribnting this circumstance to the instrument used, the first thing at hand being too thick at the point. I followed up Dr. Hay's suggestion, and used an iron better adapted to reach the bleeding vessel, but with no good result. During the operation the patient started, by which the under lip was slightly burnt. And here I may mention the blood continued to flow from the lip pretty freely for several days.

Our success until the 23d was various, and on that day, if anything, the hæmorrhage was worse, and accompanied by alarming symptoms, with weak pulse, giddiness, &c. I had serious thoughts it would be necessary to take up the carotid. Towards evening an improvement took place, the bleeding being once more under command by pressure, &c. Mild purgatives ordered, in consequence of a considerable quantity of blood having been swallowed.

At 2 A. M., of the following morning, I was sent for, as the patient had sunk to an alarming degree. Dr. Hay and myself attended immediately; we found him recovering from a fainting fit. Wine given, &c. He rallied; and upon examination found there was now no active hæmorrhage from the original source; nor was there any afterwards. In the course of the day Mr. Nasmyth, of George street, saw the case, which was going on favorably, with the exception of a tolerably smart oozing from the gums, and slight bleeding from the left nostril, which commenced after the hæmorrhage from the alveolus had become less active. Upon the removal of the bandages the face was found much discolored and swollen from the effusion of blood into the cellular tissue, giving all the appearance of the result of a blow. Pulse good; countenance less anxious; getting a quiet sleep occasionally; the sloughs drying up under the use of the camphorated spirit, and latterly of turpentine, with no increase of hæmorrhage. Mild aperients given; a little wine, and the use of tonics: up to the 27th, upon the whole, continuing to improve. The oozing from the gums and nostril being occasionally troublesome, a strong solution of the nitrate of silver was painted over them with advantage. At this stage Dr. Hay and Mr. Nasmyth considered it unnecessary to continue our meetings as we had done, but to see him occasionally, Dr. Hay taking charge of the case, the patient remaining much in the same state until the 7th of January, 1842.

I had not seen the case for two days, when Dr. Hay informed me that a change for the worse had taken place—all the old symptoms, aggravated by a severe pain all over the mouth and head. Mr. Nasmyth and myself saw the patient on Sunday, the 9th, the third from the removal of the tooth, and found him much reduced; the gums turgid to a remarkable degree, and of a deep purple color, almost covering the teeth, and bleeding freely; the blood was again oozing from the alveolus, and slightly from the nostril; the features collapsed; complained of blindness; and all the symptoms of the disease "purpura hæmorrhagica" more decided. Mr. Nasmyth employed a solution of the proto-nitrate of mercury to the gums, which only checked the hæmorrhage for a short time. Wine (claret) given freely, stimulants, tonics, &c.

On Sunday, the 9th, Dr. Abercromby was consulted; but although all was done that such eminent men would be expected to do, death put an end to this painfully-interesting case on the Tuesday following, being three weeks and two days in duration.

In the course of my practice I have met with several cases of severe hæmorrhage following the extraction of a tooth, but have always succeeded, with the exception of the above case, in arresting it by pressure. In one case in particular the hæmorrhage was alarming. Upon examining the mouth, I discovered a portion of the alveolar process that had been splintered; upon removing this and the clot which nearly filled the mouth, and, in fact, was acting as a poultice, and washing out the bleeding alveolus with warm water, I cut a small piece of sponge tent nearly to the size of the cavity, and pressed it firmly down with lint; over that a compress of cork, and all securely bandaged, with complete success. The heat of the mouth softens wax; but sponge expands, and being confined, must of necessity press upon the mouth of the bleeding vessel. I have occasionally tried replacing the tooth with lint wrapped round the fangs, but never could depend upon it, but should think it would answer well with any of the single-rooted teeth, or bicuspids: I never had occasion to try it in any of these teeth. In passing I may remark, that in all the cases that have come under my notice, I never saw the application of the actual cautery of much service; still in extreme cases we are bound to employ it.—*Boston Med. and Surg. Journal*, from the *London Lancet.*

